# Toward an understanding of motivational influences on prospective memory using value-added intentions

**DOI:** 10.3389/fnhum.2015.00278

**Published:** 2015-05-18

**Authors:** Gabriel I. Cook, Jan Rummel, Sebastian Dummel

**Affiliations:** ^1^Department of Psychology, Claremont McKenna CollegeClaremont, CA, USA; ^2^Department of Psychology, Heidelberg UniversityHeidelberg, Germany

**Keywords:** prospective memory, intention, motivation, value-added, loss, reward

## Abstract

This study examined *value-added intentions* by manipulating the cognitive frame associated with monetary contingencies for detecting prospective memory (PM) cues. We associated a loss-frame with a monetary punishment for failing to respond to cues and a gain-frame with a monetary reward for remembering to respond to cues and compared those frames to a no-frame control condition with no contingency linked to performance. Across two experiments, we find increased PM performance for participants in the loss-frame (Experiments 1 and 2) and in the gain-frame (Experiment 2) conditions relative to the no-frame condition. This value-related improvement in PM was not accompanied by a significant increase in cue monitoring as measured by intention-induced interference to an ongoing task and recognition memory for ongoing-task items. The few previous studies investigating motivational PM showed mixed results regarding whether PM improves due to incentives or not. Our results provide further evidence that, under some experimental conditions, PM improves with rewards and that the benefit generalizes to penalizing performance. The results have both practical implications and theoretical implications for motivation models of PM.

## Introduction

Prospective memory (PM) is a cognitive ability which enables individuals to remember to execute an intended action plan at the appropriate moment in the future (e.g., Ellis and Kvavilashvili, 2000). Typical everyday examples of PM intentions include remembering to return a book to the library on its due date or to file one’s tax return within the deadline. As evident from these examples, PM intentions are likely to be forgotten entirely or delayed until a later time in the future. On the one hand, PM failures may be associated with additional charges like late fees or service fees. On the other hand on-time PM fulfillment may be rewarded with monetary incentives, like an additional bonus for keeping the deadline. Such monetary charges and rewards are meant to increase the motivation not to forget pending intentions but we are unsure whether they are actually effective at increasing the likelihood of intention fulfillment. We designed the present research to address this question and to develop a better understanding of the general relationship between such motivational and cognitive processes in the PM domain.

Einstein and McDaniel (1990) introduced a task set that allows studying PM in the laboratory. At the outset of the task, participants first form the action plan to execute a certain action (e.g., to press a special key) in response to a target cue which occurs during a later to-be-performed ongoing task. After some distractor activity, participants then perform the ongoing task and meanwhile they have to remember on their own to fulfill the intended action in response to PM cues. This task set is meant to simulate the typical PM situation of being actively engaged in an ongoing activity and nevertheless having to remember to fulfill intentions at the appropriate moment. Critically, PM performance can be measured as the proportion of correct responses to PM cues. This task set has been used extensively to study the cognitive processes underlying PM abilities. Based on findings that successful PM performance often comes at a cost to currently ongoing tasks (e.g., slowed responding; Marsh et al., 2003; Smith, 2003), cognitive theories of PM assume that attentional resources are regularly recruited to monitor the environment for the occurrence of PM cues (McDaniel and Einstein, 2000, 2007; Smith, 2003, 2010). According to a multi-process view of PM, however, PM performance cannot only rely on effortful attentional processing, but also on more effortless processes when PM cues spontaneously triggers intention retrieval (McDaniel and Einstein, 2000, 2007; Einstein et al., 2005).

Whereas there is a large body of empirical studies investigating the cognitive processes underlying PM, only few studies have investigated motivational PM processes. This lack of empirical investigation for how motivational states affect PM is especially surprising because it is of high face validity that the motivation to fulfill an intention may influence actual intention fulfillment.

In an attempt to link motivational and cognitive PM processing, Penningroth and Scott (2007) recently suggested a *goal-based* *motivational-cognitive model*. According to this framework, motivation to fulfill an intention increases whenever an intention becomes relevant for one’s personal goals and will therefore influence encoding, maintaining, and retrieving of the intention. This argument is built on the idea that people place a special cognitive effort on intentions that are personally relevant, perhaps because goal-relevant intentions are perceived as especially important. Perceived importance, in turn, has been shown to affect PM fulfillment. In a correlational diary study, Andrzejewski et al. (1991) found that participants reported remembering more intentions that they perceived as more important (see also Penningroth and Scott, 2007). Similarly, experimental studies have shown PM improvements when instructions stressed the importance of the PM task relative to the ongoing task (Kliegel et al., 2001, 2004). Often, these improvements come at the cost of greater interference with the ongoing task (Smith and Bayen, 2004; Marsh et al., 2005; Loft and Humphreys, 2012; see also Walter and Meier, 2014, for a comprehensive review of importance effects). Important intentions seem to induce a stronger engagement in effortful *attentional monitoring* for PM cues when it is time to fulfill the intention (i.e., described as late PM-processing stages; Penningroth and Scott, 2007).

Further, the goal-based motivational-cognitive model suggests that personal relevance of an intention can affect intention encoding (i.e., described as early PM-processing stages). In detail, goal-relevant intentions are assumed to become *better accessible* in memory and/or to encourage the *use of better intention-encoding strategies*. These early processes are assumed to facilitate a spontaneous intention retrieval triggered by the PM cue (cf. Einstein et al., 2005). In support of these assumptions, Penningroth and Scott (2007) find that individuals retrieved everyday intentions earlier in a recall test and also used strategies more frequently, when intentions are personally relevant. With a younger population, Somerville et al. (1983) showed that children who had a high-interest intention to remind their caretakers to purchase candy remembered to do so more than those who had a low-interest intention to remind their caretakers to retrieve the laundry. Thus, intentions with high personal relevance seem to change motivation to remember future intentions.

Empirical support for the assumptions that cues for goal-relevant intentions are highly accessible comes from findings that PM cues that are emotionally laden are better detected than neutral cues without additional attentional effort (Clark-Foos et al., 2009; Marsh et al., 2009; Altgassen et al., 2010; Schnitzspahn et al., 2012) and that items that are related to personal goals have a retrieval advantage (Förster et al., 2005; Penningroth and Scott, 2007).

The assumption that strategy use can indeed change later PM processing is supported by research on implementation-intention encoding strategies of PM. Implementation intentions are specific *when-then* plans that associate an intention with an action and are usually expressed in the form “*When situation X occurs, then I will perform response Y!”* (Gollwitzer, 1999, p. 494). The specificity of encoding these intentions, compared with less specific encoding, improves PM performance in both laboratory settings (Cohen and Gollwitzer, 2008; McDaniel et al., 2008) and naturalistic settings (Liu and Park, 2004). Implementation intentions benefit goal-striving behavior (Gollwitzer, 1999; Gollwitzer and Sheeran, 2006), but the underlying cognitive mechanisms for explaining the benefit have only recently been better understood. One prevailing theory for this benefit is that implementation-intention encoding encourages cue accessibility and spontaneous retrieval (McDaniel et al., 2008; McDaniel and Scullin, 2010). Another theory is that cue detection for implementation-intentions results from stronger engagement in attentional cue-monitoring (Meeks and Marsh, 2010; Zimmermann and Meier, 2010, for a similar finding) Support for these theories, however, may be contingent on the processing demands of the intention studied (e.g., whether cues are focal to the ongoing task or not). Rummel et al. (2012) set out to test the cognitive underpinnings of implementation intentions by using appropriate control conditions and found evidence that both implementation-intention encoding and increased cue-activation encoding indeed foster spontaneous retrieval processes. This finding might be relevant for the understanding of motivational PM processing, because according to Gollwitzer (1999), when we engage goal-striving cognitive processing, remembering to carry out important intentions improves. When not explicitly instructed, the willingness to seek out goal-striving behaviors spontaneously and engage in useful encoding processes (viz., implementation-intention or cue-activation encoding) may be one of the motivational differences.

In sum, according to the goal-based motivational-cognitive model suggested by Penningroth and Scott (2007), successful PM performance is reflected by the cognitive processes that operate throughout the PM task; the model does not seem to argue whether effortful attentional or spontaneous processing is necessarily better for PM performance, but it clearly suggests that motivational PM processing can recruit both types of cognitive processes to ensure good PM performance. Their model is nice insofar as it incorporates research from the goal literature (Kruglanski et al., 2002; Förster et al., 2005; Kruglanski and Kopetz, 2009) as well as the PM literature already discussed, especially in relation to the distinction between effortful vs. effortless retrieval of intentions (Einstein and McDaniel, 2010; Smith, 2010). Like Penningroth and Scott (2007), we believe that motivational variables are important to consider when studying PM both in the laboratory and in naturalistic settings and we find their model to be fruitful in guiding motivation research on PM.

Since Penningroth and Scott (2007) introduced their model, unfortunately very little experimental research has set out to test and validate its predictions. One exception is a recent study by Brandimonte et al. (2010) who showed that associating a PM intention with the pro-social goal to “*help the experimenter to get good data*” resulted in better PM performance compared to a standard PM task (see also D’Angelo et al., 2012). Another widely used method to increase personal relevance of a task and thus to motivate good performance is to change the value of the task (Atkinson, 1964). Typical value manipulations are monetary punishments for bad performance or rewards for good performance. Such manipulations have been shown to improve performance in attention tasks (Hübner and Schlösser, 2010; Wentura et al., 2014), encoding of episodic memories (Shigemune et al., 2014), and even accessibility of personal goals (Förster et al., 2005). Importantly, there is empirical evidence that already small financial incentives can change the motivation to perform a task (Locke and Braver, 2008; Chiew and Braver, 2011; Marien et al., 2013, 2014).

There are also a few studies that have tested whether the effects of rewards on some cognitive domains generalize to PM performance (Furst, 1986; McCauley et al., 2009, 2011; Brandimonte et al., 2010). McCauley et al. (2009) manipulated monetary incentives to influence PM in a pediatric traumatic brain injury (TBI) population. Participants in a low-motivation condition could trade points earned by responding accurately to PM cues for pennies, whereas participants in a high-motivation condition could trade points for dollars. They found that the high-motivation condition showed a greater benefit on PM compared to the low-motivation condition; this benefit was present in all groups of children with TBI. In a later study, McCauley et al. (2011) used a different motivation manipulation and found that participants with moderate TBI showed better PM in the context of high-motivation (dollar incentive) relative to a low-motivation (penny incentive); the greater incentive did not help participants with severe TBI. These interesting findings raise two important questions about incentives and PM. First, improved PM for those with moderate, but not severe TBI elevates the importance of whether motivation incentives can improve PM for healthy individuals. Second, the lack of a control condition in the McCauley et al. studies raises the question about whether the low-motivation conditions improved PM beyond having no incentive at all, especially for healthy individuals. However, in the only study that used a reward manipulation with a healthy student sample and used a no-reward control condition, Brandimonte et al. (2010) found that rewards in terms of extra course credit did not result in better PM performance. In fact, the reward even reduced performance in the pro-social condition, but not in the standard PM condition. This deficit might be due to the reward changing the nature of social relationships (Fehr and Rockenbach, 2003; Heyman and Ariely, 2004). Taken together, the results are mixed regarding whether manipulation of the personal value of an intention affects PM performance. Further, the goal of the first two studies focused on providing remediation of PM impairments for individuals TBI rather than outlining any potential cognitive mechanism underlying motivational effects. None of these three studies attempted to understand the cognitive dynamics associated with improvements due to increased value of intention fulfillment.

We assume that there are many reasons that motivate individuals to come up with a certain intention in the first place (cf. Kruglanski et al., 2002). The aim of the present research, however, is not to investigate the motivation behind the selection of a specific intention among all possible intentions but to investigate differences in the motivational value of intention fulfillment. Our reasoning is that the very same intention can vary widely in personal value depending on the situation or circumstances. The execution of an intention may sometimes be of lower value (e.g., the intention to make a cup of coffee as part of the breakfast routine) and sometimes of higher value (e.g., the intention to make a cup of coffee to wake oneself up after a poor night’s sleep, making a cup of coffee to accommodate guests who stayed over night). Our goal was to highlight the role that such value added to an intention can play in encoding, maintaining, or retrieving intentions. Although our study is laboratory based, we assume many of the same, and likely more, motivational influences will shape real-world intentions in similar ways.

Scholars of PM have not yet developed any working theories regarding the motivation involved in how we execute intentions related to incentives. We have chosen to introduce the term *value-added intentions* to identify a general class of intentions that are associated with either the attainment or the forfeiture of some entity or concept possessing some personal value over and above the value of getting something done that one had planned to do. In everyday life, such additional value can come from intention-related rewards or penalties that are either tangible or conceptual and have economic value as with money, points, or objects (e.g., gains or losses), social value (e.g., acceptance/praise or rejection/criticism), or physiological value (e.g., hunger satiation or food deprivation).

In the laboratory, value-added intentions may be defined as intentions that possess some value over and above the value of being compliant with instructions. Such value-added intentions can be realized by associating intention fulfillment or failure with some kind of personal consequence for the participant.

We believe that value-added intentions can be distinguished from standard intentions that are generally associated with no consequence, so that there is no additional motivational incentive to fulfill them. Perhaps value-added intentions operate in much the same way as other intentions, which might desensitize any appeal for investigating them in the laboratory. Alternatively, value-added intentions may operate like important intentions (which may or may not have consequential value) and require more attentional resources (Smith and Bayen, 2004; Marsh et al., 2005). Finally, value-added intentions might elicit better intention-encoding strategies and thus operate like implementation intentions and automatize intention fulfillment (Rummel et al., 2012) or render intention-related items more accessible because they have a special meaning for the individual (cf. Förster et al., 2005). Because the field of PM has not studied value-added intentions in any depth, possible theories that researchers develop about the processing of these intentions are tentative and untested. The absence of compelling research or guiding framework for value-added intentions does not seem to be good reason for not studying intentions that have great personal and economic impact.

Our goal of this set of experiments was to investigate how individuals remember value-added intentions, which we chose to study by associating intentions to financial gains and losses.

## Experiment 1

Previous research has revealed favorable benefits of monetary gains on PM for groups of participants with cognitive impairments (McCauley et al., 2009, 2011). To our best knowledge, however, no prior study has investigated the effects of monetary incentives in healthy populations. In everyday life, there are different types of intentions with monetary consequences. Sometimes intention fulfillment is associated with monetary rewards; sometimes intention failure is punished with fees. As there is no prior research on the effects of financial losses on PM, we started with examining whether associating a financial loss with failing to remember an intention would improve intention fulfillment in Experiment 1. In Experiment 2, we then additionally considered effects of financial gains associated with successful intention fulfillment.

As an overview of the methodology, we used a standard laboratory PM task setting with a lexical-decision task as ongoing task and the PM task to respond to items containing a special syllable by pressing a designated key on the keyboard. Further we paid all participants prior to instruction, but manipulated the cognitive frame associated with the payment. Participants in the control condition (no-frame) received their payment independent of their task performance, whereas participants in the loss-frame condition received payment contingent on their PM performance. Importantly, by paying *all* participants at the outset of the experiment, we ensured that participants would experience a PM-performance-contingent payment reduction as a true loss of money they already possessed rather than as a lost opportunity to gain money.^1^ In doing so, we aimed to create a situation most similar to receiving a fee for PM failures as is common outside the laboratory.

We can use the cognitive pathways outlined in the motivational-cognitive model (cf. Penningroth and Scott, 2007, Figure 1) to help guide some predictions about how value frames might change goal relevance and ultimately PM processing. According to the model, the loss frame might cause participants to adopt different encoding strategies. Any observed PM improvement associated with the loss frame would reveal evidence for more automatic retrieval of the intention as indicated either by equated, or even reduced, ongoing-task costs. Similarly, increased intention values might foster intention accessibility, which Penningroth and Scott propose would also provide evidence for more automatic influences on PM. In contrast, if the loss frame increases goal relevance by increasing attentional effort toward the PM task relative to the no-frame condition, they would expect an increase in PM accuracy, which would also come at a performance cost to the ongoing task.

### Method

#### Participants and Design

We recruited 66 student participants from Heidelberg University (mean age: 22.77; range: 18–35; 79% female) who were all native speakers of German. We assigned an equal number of participants randomly to two experimental conditions and had them complete 30-min test sessions in groups with up to six participants per session. We excluded from all analyses one participant who did not follow task instructions and one participant who received an incorrect amount of starting money accidentally.

All participants received 4€ starting money. In one condition (loss-frame condition; *n* = 32), participants lost a certain percentage of this money, whenever they forgot to press the PM key in response to a PM cue. In the other condition (no-frame control condition; *n* = 32), PM failures were not tied to a monetary loss. PM performance was compared between the two groups. We used the computer program G*Power to calculate the statistical power (Faul et al., 2007). The power to detect medium-sized PM performance differences was moderate, 1 − β = 0.50. All participants performed two blocks of an ongoing lexical-decision task; in the first block they performed the ongoing task alone (baseline) and in the second block they had an additional PM task to perform (PM block). This resulted in a 2 by 2 mixed design for ongoing-task performance with the between-subjects factor condition (loss, control) and the within-subjects factor block (baseline, PM block). The power to detect loss-induced differences in changes in ongoing-task performance from the baseline to the PM block of medium size was excellent, 1 − β > 0.99.^2^

#### Materials and Procedure

For an ongoing lexical-decision task, 240 words of medium length and frequency were taken from a word-norm German database (Heister et al., 2011). We used half of the words to create pronounceable nonwords by swapping one to two letters. Importantly, these words and nonwords were restricted not to contain the syllable *ung* because this syllable served as cue for the PM task. For the PM task, 12 additional words of comparable length and frequency with the syllable *ung* were taken from the same database, 6 of which were transformed into nonwords. The PM-cue words and nonwords were *Haltung, Wirkung, ungefähr, ungern, Zungen, Jungen, Sölung, Trüspung, Ungran, tungeln, ungren*, and* Daunga*.

Both experiments reported here were conducted in accordance with national ethical guidelines. We recruited participants from campus and promised them a chocolate bar for their participation. After providing informed consent, participants were seated in cubicles and in front of a computer screen and then received an envelope containing 4€ and a note with either the number “1” or “2” on it from the experimenter. The experimenter was not aware which number was in a given envelope. The experiments’ starting screen prompted participants to enter the number from the envelope and by doing so participants assigned themselves to one of the two experimental conditions (loss-frame, no-frame). Participants were next asked to enter the amount of money they found in the envelope and to then put the money and the note back in the envelope and the envelope into a box which was located on the side wall of the cubicle outside of participants’ field of view.

Next, all participants received instructions for the ongoing lexical-decision task asking them to press the *J*-key for words and the *F*-key for nonwords. Then, all participants performed a baseline block of 80 lexical-decision trials. For this baseline block, the computer software selected a random subset of 40 words and 40 non-words from the set of 240 word and nonword items for each participant anew. Items were presented randomly intermixed in white font on a black background. Each trial started with a fixation cross of random duration (250–700 ms) followed by an item which remained on the screen until participants responded to it. A blank screen separated trials for 500 ms.

After the baseline block, participants received instructions on the screen that they would perform another block of the lexical-decision task later on. For this later block, all participants received the additional PM-task instruction to respond to all items (i.e., words and nonwords) containing the syllable *ung* or *Ung* by pressing the *hyphen*-key (note that the *hyphen*-key on the German QWERTZ keyboard is located at the position of the *slash*-key on a QUERTY keyboard). Participants were instructed that they should press the *hyphen*-key instead of the word/nonword classification on *ung*-trials but that late *hyphen*-key presses after having pressed another key would still be counted as correct. At this point, all participants were also informed that they would receive (some of) the money from the envelope as additional compensation for their participation. Only participants in the loss-frame condition, however, received additional instructions that the money from the envelope was the maximum amount of money (starting money) they could receive and that they would lose some part of this money whenever they forgot to press the *hyphen*-key for *ung*-items. Importantly, participants were *not* informed how many *ung*-items would occur during the task. Instead they were made aware that their final compensation was contingent on their overall performance in the PM task and that their starting money would be proportionally reduced whenever they missed a *ung*-item based on the percentage of cues they missed. To assure that participants understood these instructions, they were further informed that they could receive the complete amount of money in the envelope if they never missed a *ung*-item but also lose all the money if they never responded to a *ung*-item correctly. In the control, condition participants were simply informed that they would receive the money from the envelope after the experiment.

Participants of both conditions then answered trivia questions for 4 min to delay the PM task. After this filler task, participants performed the second lexical-decision-task block with the embedded PM task (*PM block*). This block comprised 172 trials in total; 80 word trials and 80 nonword trials, to which the remaining 160 items from the item set were assigned randomly, as well as 12 PM trials. PM trials occurred on fixed positions for all participants (i.e., on Trials 20, 30, 43, 55, 66, 80, 92, 105, 116, 128, 138, and 150) and *ung*-items were assigned randomly to these trials.

After the PM block, participants’ memory for the PM cues (syllable *ung*) and the PM key (*hyphen*-key) was probed. Finally, participants were debriefed and received the chocolate bar promised to them when they were recruited and the monetary compensation, which was promised to them in the course of the experiment. For participants in the loss-frame condition, monetary compensation was proportionally reduced contingent on their PM performance.

### Results and Discussion

We set an alpha-level to 0.05 for all analyses. We further report Bayes factors (BF) for those results that are most critical for our conclusions. In line with conventions, we applied the JZS prior (Jeffreys, 1961). Note that we report BF in favor of the evidence (i.e., BF for the alternative hypothesis (BF_10_) when evidence is in favor of differences and BF for the Null hypothesis (BF_01_) when evidence is in favor of no difference). Thus, BF values greater than one always reflect our interpretation of the data.

#### Prospective-Memory Performance and Intention Memory

The proportion of correct responses to PM cues was used to assess PM performance. Any *hyphen*-key press between PM cue onset and the offset of the probe stimulus on the following trial was counted as a correct PM response. See Table 1 for means and standard errors.

**Table 1:** **Prospective-memory and ongoing task performance in Experiment 1**.

	PM Performance		Ongoing-Task Performance
	PM Hit Rates	Error Rates	Word RTs	Nonword RTs
**Loss**
Baseline		0.034 (0.007)	854 (92)	986 (82)
PM Block	0.79 (0.04)	0.030 (0.003)	1042 (70)	1184 (86)
*PM Costs*		−0.004 (0.005)	188 (55)	198 (55)
**Control**
Baseline		0.039 (0.006)	733 (36)	867 (64)
PM Block	0.65 (0.05)	0.034 (0.005)	878 (45)	970 (58)
*PM Costs*		−0.005 (0.005)	144 (25)	103 (32)

*Note. PM = Prospective memory, RT = response time in ms. Baseline = first block without PM task, PM Block = second block with PM task. For the PM Costs measure, performance in the baseline block was subtracted from performance in the second block. Standard errors are presented in parentheses*.

An independent-samples *t*-test was used to compare PM performance between the loss-frame and the no-frame condition. This test showed that PM performance in the loss-frame condition (*M* = 0.79; *SE* = 0.04) was significantly better than in the no-frame condition (*M* = 0.65; *SE* = 0.05), *t*_(62)_ = 2.03, *d* = 0.52, *p* = 0.047 (BF_10_ = 1.42).^3^ To our best knowledge, this result is the first empirical demonstration that anticipated monetary losses associated with a PM failure can cause PM improvements.

All participants were able to recall the PM key after the experiment. Only two participants were unable to recall the PM cue and these two participants nevertheless made at least one correct PM response during the ongoing task. Therefore, PM encoding failures or retrospective forgetting of the PM task cannot account for the present findings.

#### Ongoing-Task Performance

Lexical-decision error-rates and response times (RTs) were used to assess ongoing task performance (see Table 1 for means and standard errors).

Error rates were submitted to a 2 × 2 mixed-model analysis of variance (ANOVA) with the between-subjects factor condition (loss-frame, no-frame) and the within subjects-factor block (baseline, PM block). There were neither significant main effects nor an interaction for error rates, all *F*s < 1.6. This result is not surprising because error rates were near ceiling in all conditions, which is typical when using a lexical-decision task as ongoing task (cf. Brewer, 2011).

RT analyses were confined to correct responses and the first four trials as well as the four trials after a PM trial were excluded to account for artificial costs or after-effects associated with these trials (Brewer, 2011; Meier and Rey-Mermet, 2012). Responses faster than 300 ms and slower than two standard deviations above the individual mean were excluded. RTs were trimmed separately for each block and item type (word, nonword). RTs were then submitted to a 2 × 2 × 2 mixed-model ANOVA with the between-subjects factor condition (loss-frame, no-frame) and the within subjects-factors block (baseline, PM block), and item type (word, nonword). This analysis showed a non-significant trend of generally slower RTs in the loss-frame (*M* = 1016; *SE* = 64) than in the no-frame condition (*M* = 862; *SE* = 64), *F*_(1,62)_ = 2.97, *f* = 0.22, *p* = 0.090. Further there was a main effect of block, *F*_(1,62)_ = 30.99, *f_z_* = 0.27, *p* < 0.001,^4^ indicating that responses were generally faster in the baseline (*M* = 860; *SE* = 48) compared to the PM block (*M* = 1018; *SE* = 46). This result is in line with the typical finding that the addition of the resource-demanding PM task comes at a cost to performing the ongoing task (Einstein and McDaniel, 2010). We cannot rule out that the main effect of block might have been due to a general fatigue effect (Smith, 2010), but the present design was tailored to test for differences in task interference due to a monetary loss associated with PM failures. In this regard, the critical finding is that the trending effect of condition and the effect of block were not further qualified by an interaction between condition and block, *F*_(1,62)_ = 1.48, *f_z_* = 0.06, *p* = 0.229. Notably, this interaction test had a very high statistical power and the non-significant finding can be seen as evidence that the differences in PM performance between the loss-frame and the no-frame condition were not accompanied by equivalent differences in the engagement in PM monitoring. For a better illustration of these findings, we computed difference scores by subtracting baseline RTs from PM-block RTs (see Smith and Bayen, 2004, for a similar approach). The cost difference scores were numerically higher in the loss-frame condition than in the control condition for both words and nonwords (see Table 1). However, the differences in PM-induced costs between the two conditions were not statistically reliable for either words, *t* < 1 (BF_01_ = 3.15), or nonwords, *t*_(62)_ = 1.48, *d* = 0.38, *p* = 0.143 (BF_01_ = 1.55).

The only other significant result was a main effect of item type, *F*_(1,62)_ = 28.23, *f_z_* = 0.26, *p* < 0.001. This finding shows that participants responded to words (*M* = 878; *SE* = 43) faster than to nonwords (*M* = 1002; *SE* = 49) which is also a typical finding when using lexical-decision tasks (all other *F*s < 1.1).

In sum, results of Experiment 1 are the first demonstration that monetary punishment of PM failures is effective in improving PM performance. There was no reliable support, however, for the hypothesis that these PM improvements are accompanied by higher engagement in effortful PM processing as reflected by higher PM-induced costs.

## Experiment 2

Experiment 1 revealed that framing PM forgetting in terms of a loss increased PM performance, but this benefit was not offset by a significant increase in response latencies on the ongoing task. Our results are intriguing because they seem to show that certain framing manipulations (i.e., a loss frame) can potentially increase PM. Furthermore, these PM improvements are unlikely to be fully explained by an additional allocation of attention to the PM task, because, despite good statistical power, the small numerical loss-induced increase in costs to the ongoing task was not significant. Because this experiment is the first to show this finding, we believe it prudent to replicate this finding.

In order to better understand how individuals fulfill value-added intentions, we decided to broaden our frame manipulation by adding a gain-frame condition to the design. Previous investigations of monetary incentives on PM (McCauley et al., 2009, 2011) did not investigate the attentional demands of intentions using monetary incentives. There is reason to believe that improved PM performance using a gain-frame might be due to greater allocations of attention toward the intention itself. Numerous studies have found slowing on an ongoing task when paired with fulfilling various types of intentions (Marsh et al., 2003; Smith, 2003, 2010). Although this attentional tradeoff may not be important for questions designed to improve PM for patients with TBI, understanding attentional demands might be relevant for intentions that are maintained or carried out in more fast-paced or attention-demanding environments (e.g., hospitals, air traffic control, driving, etc.; cf. Dismukes, 2012; Grundgeiger et al., 2014) and also in everyday life where maintaining good ongoing task performance without forgetting intentions is crucial. Critically, cases of everyday intentions are also qualified by incentives or disincentives. We often have intentions to cash a sizable tax return or to retrieve a paycheck from our boss. In contrast, we may also need to remember to pay a bill before a due date or we may need to return money borrowed from a friend. Certainly, there are some qualitative differences between these sets of example intentions. The former two intentions involve gaining access to money not otherwise endowed and the latter two involve parting with money currently held. Research in other cognitive domains has shown that people process information differently based on whether a cognitive frame is a gain or a loss (Tversky and Kahneman, 1981; Shah et al., 1998). Therefore, it may be also of theoretical interest to test whether loss and gain frames have differential effects on value-added intentions.

Beyond these applied implications, a basic question concerning the role of effortful PM processing for value-added intentions is of theoretical interest. As our first study might not have been optimal in this regard, we made some procedural changes for the second experiment.

One potential limitation of Experiment 1 is that participants in the no-frame condition could have interpreted the payment they received as some financial gain (reward) for detecting PM cues. If this assumption were true, the difference in PM accuracy between the no-frame and the loss-frame conditions could be interpreted in ways other than we postulate. For example, if participants interpreted the no-frame condition as a gain, Experiment 1 might reveal differences between gaining and losing money. In that case, an unanticipated perception of gaining money for those in the no-frame condition would indicate that paying participants actually reduced performance relative to losing money, perhaps because of reduced motivation (see Brandimonte et al., 2010). Alternatively, if participants perceived payment in a general sense and unrelated to PM specifically, the increased performance for the loss-frame condition would indicate losing money changed performance in a positive way. Adding a “true” gain-frame condition, however, should eliminate ambiguity about the results.

Evidence against the criticism just raised is that several participants of Experiment 1 also told the experimenter after the experiment that they thought that the information about monetary incentives was fictional and that they were surprised that they actually got paid. We did not consider asking this question, so we cannot provide an accurate representation of this outcome. The reason for this assumption seemed to be that during recruitment we told participants that they would get a chocolate bar for participating, but we made no mention of compensation. Because this confusion likely weakened the effect of the loss manipulation, participants of Experiment 2 were informed during recruitment that they would receive a monetary compensation for their participation and that compensation could vary depending on their performance.

Furthermore, we made three experimental changes to this experiment. Two additional tasks were applied after the PM task to shed light on the processes underlying the loss-induced PM benefits. One task was a surprised recognition test for ongoing-task items of the PM block. The idea behind this task is that a more careful ongoing-task processing under the loss-frame (and eventually also a gain-frame) conditions should be reflected by better item recognition (see Loft and Humphreys, 2012, for a similar task and rationale). The other additional task was a Go/No-Go task in which go and no-go trials were indicated by two different font colors. The response key for go-responses was the *hyphen*-key (which served as PM key in the previous lexical-decision task) and the probe stimuli were the PM cues as well as some neutral ongoing-task items from the previous lexical-decision task. With this additional task, we aimed to test whether the intention becomes more accessible for participants in the frame condition compared to the no-frame control condition. As the intention during the preceding lexical-decision task was to respond to PM cues with the *hyphen*-key, faster responses on PM-cue go-trials compared to neutral-item go-trials in the frame conditions relative to the no-frame would indicate that the intention (i.e., *press hyphen-key for PM cues*) is more accessible in these conditions (cf. Förster et al., 2005, for a similar rationale). Based on this rationale, one could even expect more no-go errors on PM-cue no-go trials than on neutral-items no-go trials. This modification helped us to address whether value adding manipulations might increase the accessibility of the intention, a component of the Penningroth and Scott (2007) model. Finally, after completing the experiment, participants indicated the importance of the ongoing task and the PM task to test whether perceived importance of the two tasks would change with the value frame. These questions were also inspired by the Penningroth and Scott model which assumes that motivation-induced PM improvements are due to higher perceived importance of goal-related intentions.

### Method

#### Participants and Design

Eighty-five students from Heidelberg University (mean age: 22.60; range: 18–32; 79% female) who were all native speakers of German participated for monetary compensation. Participants were assigned randomly to three experimental conditions with the constraint that participants were distributed equally between conditions. One participant who did not follow task instructions and one participant who was unable to recall the PM cue and the PM key in the intention memory test and additionally never made a correct PM response were excluded from all analyses.

As in Experiment 1, all participants performed two blocks (baseline, PM block) of an ongoing task and the between-subjects manipulation was applied after the first block. In one experimental condition, PM failures were punished with monetary losses (loss-frame condition; *n* = 27). In a second condition, successful PM fulfillment was rewarded with a monetary gain (gain-frame condition; *n* = 28). The third condition was a control condition in which monetary compensation for participation was not tied to PM performance (no-frame condition; *n* = 28). The statistical power to detect medium-sized PM performance differences was moderate,1 − β = 0.50. The statistical power to detect medium-sized differences in ongoing-task performance changes from the baseline to the PM block, however, was excellent, 1 − β > 0.99.

#### Materials and Procedure

Materials were the same as in Experiment 1. Participants were again recruited on campus. This time, however, participants were informed already at recruitment that they would receive a monetary compensation for their participation which would vary between 5€ and 9€ contingent on their performance. When entering the laboratory, participants were seated in cubicles and then first participated in an unrelated questionnaire study for 20 min before they started with the 30-min PM experiment.

As in Experiment 1, participants provided informed consent and then received lexical-decision task instructions and performed a baseline block first. For the PM block, participants received the same additional PM instructions as in Experiment 1. After PM instructions, participants were asked to take an envelope from the box at the side wall. There were different envelope versions for the three conditions. As in Experiment 1, the envelope versions for the loss and control conditions contained 4€ and a note with the number “2” or “3”, respectively. For the gain-frame condition, the envelope only contained a note with the number “1”; the envelope contained no money. Participants were prompted to enter the number and the amount of money they found in the envelope on the computer screen. By entering the number, participants assigned themselves to the experimental conditions thus experimenters were blind to which condition a given participant was assigned. Participants were then asked to put the note and money back into the envelope and the envelope back into the box. Next, participants were informed about the money they could possibly earn and this information varied with conditions. In the loss-frame condition, participants were informed that they would receive the additional 4€ from the envelope in addition to the 5€ basic payment, but they would lose a certain percentage of this money contingent on the percentage of *ung*-items they would miss. In the gain-frame condition, participants were told that they could earn up to 4€ in addition to the basic payment of 5€ and would gain a certain percentage of money contingent on the percentage of *ung*-items they would respond to correctly. In the no-frame control condition, participants were simply told that they would receive the 4€ from the envelope in addition to the basic payment of 5€ as compensation for their participation. After a filler task (cf. Experiment 1), all participants performed the PM block, which was identical to the one used in Experiment 1.

After finishing the PM block, participants were presented with a surprise recognition test for ongoing-task items. For this task, 20 items (half words, half nonwords) were chosen randomly from the body of items presented in the PM block of the ongoing task and 20 new items (half words, half nonwords) that matched the other items on both usage frequency and letter length were drawn from the same database (Heister et al., 2011). None of these items contained the PM-cue syllable. Items appeared sequentially in a random order. For each item, participants indicated whether it had occurred during the lexical-decision task or not.

All participants also performed a Go/No-Go task in which the 12 PM cues and 12 neutral ongoing-task items from the PM block served as probe stimuli. Stimuli for this task appeared in a random order and initially in white font color until 500 ms had elapsed and the color changed to either blue or green. Participants’ task was to press the PM key (*hyphen*-key) as fast as possible when the font color turned to blue (or green), but not to respond when it turned to green (or blue). The assignment of colors to Go/No-Go responses within each condition was approximately counterbalanced. The proportion of go relative to no-go trials was 2:1. There was a window of 1500 ms to respond to a stimulus and the inter-trial interval was 1000 ms.

After the Go/No-Go task, participants had to recall the PM cue and the PM key and were asked to indicate (on a scale from 0 to 100) how important they perceived the lexical-decision task while performing the PM block; how important they perceived the PM task while performing the PM block; and how important they perceived the PM task relative to the lexical-decision task (0 = only lexical-decision task important; 100 = only PM task important). Finally, participants received the promised monetary compensation and were debriefed and dismissed.

### Results and Discussion

#### Prospective-Memory Performance and Intention Memory

As evident from Table 2, PM performance of Experiment 2 was generally better than Experiment 1. This benefit might be due to the fact that all participants were promised monetary compensation for participation when they were recruited. A one-way between-subjects ANOVA comparing PM performance between the loss-frame, gain-frame, and no-frame conditions indicated that PM performance varied significantly with conditions, *F*_(2,80)_ = 6.66, *f* = 0.41, *p* < 0.002. *Post hoc* tests further revealed that PM performance in both the loss-frame (*M* = 0.93; *SE* = 0.02) and in the gain-frame (*M* = 0.93; *SE* = 0.02) conditions was significantly better than in the no-frame condition (*M* = 0.77; *SE* = 0.05), both *p*s = 0.002 (both BF_10_ > 5.39). PM performance between the loss-frame and the gain-frame conditions did not differ, *p* = 0.993 (BF_01_ = 3.68). Thus, results of Experiment 2 replicated the central finding of Experiment 1 that anticipated monetary losses associated with PM failure can result in PM improvements. Furthermore, results suggest that PM performance also benefits from monetary rewards associated with PM fulfillment (see also McCauley et al., 2009, 2011). This finding stands in contrast to previous studies in which successful PM fulfillment was also bound to a personal reward but did not result in any PM improvements in a healthy population (Brandimonte et al., 2010). We will discuss differences between these studies and ours in the General Discussion section.

**Table 2:** **Prospective-memory and ongoing task performance in Experiment 2**.

	PM Performance		Ongoing-Task Performance
	PM Hit Rates	Error Rates	Word RTs	Nonword RTs
**Loss**
Baseline		0.016 (0.004)	790 (48)	921 (60)
PM Block	0.93 (0.02)	0.016 (0.004)	983 (46)	1109 (53)
*PM costs*		<0.001 (0.003)	193 (32)	188 (37)
**Gain**
Baseline		0.024 (0.005)	857 (78)	972 (90)
PM Block	0.93 (0.02)	0.020 (0.003)	1116 (97)	1197 (81)
*PM costs*		−0.004 (0.004)	260 (42)	225 (49)
**Control**
Baseline		0.024 (0.004)	739 (39)	830 (44)
PM Block	0.76 (0.05)	0.024 (0.004)	899 (40)	988 (47)
*PM costs*		0.001 (0.005)	160 (30)	158 (32)

*Note. PM = Prospective memory, RT = response time in ms. Baseline = first block without PM task, PM Block = second block with PM task. For the PM costs measure, performance in the baseline block substracted from performance in the second block. Standard errors are presented in parentheses*.

All participants apart from one excluded person (see above) were able to recall the PM-cue syllable and the PM key in the intention memory test. Thus, the present PM performance results were not contaminated by PM encoding failures or retrospective forgetting of the PM task.

#### Ongoing-Task Performance

Mean error rates and RTs (trimmed as before) are displayed in Table 2. For accuracy, the 3 × 2 mixed-model ANOVA with the between-subjects factor condition (loss, gain, no-frame) and the within subjects-factor block (baseline, PM block) for error rates did not show a significant main effect or interaction, all *F*s < 1.5.

For RTs, the 3 × 2 × 2 mixed-model ANOVA with the between-subjects factor condition (loss, gain, no-frame) and the within subjects-factors block (baseline, PM block) and item type (word, nonword) for RTs did not show a main effect of condition, *F*_(2,80)_ = 2.09, *f* = 0.23, *p* = 0.130. Replicating findings from Experiment 1, however, there was a main effect of block, *F*_(1,80)_ = 102.41, *f_z_* = 0.50, *p* < 0.001, indicating that responses were generally faster in the baseline block (*M* = 851; *SE* = 36) compared to the PM block (*M* = 1049; *SE* = 36). The main effect of item type was also significant, *F*_(1,80)_ = 75.94, *f_z_* = 0.43, *p* < 0.001, showing that responses to words (*M* = 898; *SE* = 36) were generally faster than to nonwords (*M* = 1003; *SE* = 36). Despite the very good statistical power, there was once again no indication of a significant interaction between condition and block, *F*_(2,80)_ = 1.57, *f_z_* = 0.09, *p* = 0.214. All other interactions were also not significant, all *F*s < 1. To further investigate potential differences in the engagement in attentional monitoring between experimental conditions, we once again computed difference scores by subtracting baseline RTs from PM-block RTs (see Table 2) which reflect PM-induced costs. We then compared costs in both the gain and loss condition with costs in the control condition. When comparing the gain with the control condition, we found a non-significant trend of an gain-related cost increase for words, *t*_(54)_ = 1.95, *d* = 0.53, *p* = 0.057 (BF_10_ = 1.42) but not for nonwords, *t*_(54)_ = 1.15, *d* = 0.31, *p* = 0.255 (B_01_ = 2.38). When comparing the loss with the control condition, no evidence was found for a loss-related cost increase for neither words or nonwords, both *t*s < 1 (both BF_01_ > 2.92). Pooling together both the gain and loss conditions also revealed no support for greater interference compared to the control condition, both *ts*_(81)_ < 1.56, with support in favor of equated performance rather than differences (both B_01_ > 1.48). Thus, there was again no strong empirical support for the hypothesis that the PM improvements due to PM-performance contingent monetary losses or gains were accompanied by an increase in PM monitoring as reflected by PM-induced costs.

#### Ongoing-Task-Item Recognition

To assess retrospective memory for items that previously occurred during the PM block of the ongoing task, we calculated proportions of those words and nonwords which had been occurred during the ongoing task and that were correctly classified as “being from the ongoing task” (recognition hit rates) as well as the proportions of those words and nonwords that had not been occurred previously but were classified incorrectly as “being from the ongoing task” (recognition false-alarm rates) for each participant. To consider both hit rates and false-alarm rates simultaneously, we then computed a discrimination index (*Pr* = hit rates − false-alarm rates; Snodgrass and Corwin, 1988). *Pr*, recognition hit rates, and recognition false alarm rates were then submitted to separate 3 × 2 mixed-model ANOVAs with the between-subjects factor condition (loss, gain, no-frame) and the within subjects factor item type (word, nonword). See Table 3 for means and standard errors.

**Table 3:** **Ongoing task item recognition performance and perceived task importance in Experiment 2**.

	Ongoing-Task-Item Recognition						Importance Estimates		
	Hit Rates		False-Alarm Rates		*Pr*		OT	PM	OT/PM
	Words	Nonwords	Words	Nonwords	Words	Nonwords
Loss	0.70 (0.04)	0.68 (0.04)	0.40 (0.03)	0.21 (0.03)	0.38 (0.04)	0.47 (0.05)	80 (4)	91 (3)	58 (3)
Gain	0.76 (0.04)	0.71 (0.03)	0.41 (0.04)	0.17 (0.03)	0.34 (0.05)	0.54 (0.03)	75 (5)	93 (2)	65 (3)
Control	0.71 (0.04)	0.68 (0.04)	0.39 (0.03)	0.16 (0.02)	0.33 (0.04)	0.51 (0.04)	83 (4)	91 (2)	56 (3)

*Note. Pr = hit rate—false alarm rate, OT = ongoing task, PM = prospective memory, PM/OT = importance of the prospective-memory task relative to the ongoing task. Standard errors are presented in parentheses*.

The analysis of *Pr* only revealed a significant effect of item type, *F*_(1,80)_ = 22.93, *f_z_* = 0.23, *p* < 0.001, both other *F*s < 1.2. This pattern of results indicates that participants were generally better able to discriminate between previously presented and new nonwords (*M* = 0.51; *SE* = 0.02) than between previously presented and new words (*M* = 0.35; *SE* = 0.03), but the loss and gain conditions did not change discrimination rates. A similar pattern of results was found when analyzing hit rates and false alarm rates, separately. These results further corroborate the conclusion drawn based on the non-significant ongoing-task RT results that monetary punishment or rewards did not cause stronger engagement in PM monitoring or a more careful PM-cue checking compared to the control condition.

#### Go/No-Go Performance

Only very few participants made errors in the Go/No-Go task and therefore we refrained from analyzing error rates of this task. Instead we focused on the analysis of RTs on go trials. A 3 × 2 mixed-model ANOVA with the between-subjects factor condition (loss, gain, no-frame) and the within-subjects factor item type (word, nonword) and critical item (PM cue, neutral) for RTs did not reveal a significant main effect nor interaction, all *F*s < 2. Thus the results of the Go/No-Go task did not support the assumption that the loss and gain conditions strengthened the accessibility of the intention.

#### Perceived Task Importance

Absolute importance estimates for the ongoing task and the PM task as well as estimates of the importance of the PM task relative to the ongoing task were analyzed to test whether perceived PM-task importance changed as a function of a loss or gain-frame (see Table 3 for means). One-way ANOVAs for the perceived absolute ongoing-task importance and the perceived absolute PM-task importance revealed no significant differences between conditions, both *F*s < 1. Estimates of relative PM-task importance, however, varied significantly with conditions, *F*_(2,80)_ = 3.13, *f* = 0.28, *p* = 0.049. Pairwise comparisons further revealed that the perceived relative PM-task importance in the loss-frame (*M* = 58; *SE* = 3) did not differ from the one in the no-frame condition (*M* = 56; *SE* = 3), *p* = 0.557, whereas the perceived relative importance in the gain-frame condition (*M* = 65; *SE* = 3) differed from the no-frame condition, *p* = 0.018. There was further a trend for a higher perceived relative PM task importance in the gain-frame compared to the loss-frame condition, *p* = 0.077.

In sum, these results imply that PM-performance improvements due to monetary punishments were *not* associated with changes in perceived PM-task importance whereas PM-performance improvements due to monetary rewards were accompanied by an increase in the perceived importance of the PM task relative to the ongoing task.

## General Discussion

In two experiments, we manipulated cognitive frames related specifically to the value of fulfilling an event-based intention. We found an increase in PM performance for participants assigned to a loss-frame condition (Experiments 1 and 2) and to a gain-frame condition (Experiment 2) relative to participants in a no-frame control condition who also received payment for their participation, but for whom payment was not contingent on PM performance. The increase in PM for the two framing conditions was not associated with a significant increase in costs to the ongoing task. Importantly, improved PM for our gain frame replicates previous research on the benefits of monetary incentives on PM in cognitively-impaired participants (McCauley et al., 2009, 2011) with a sample of healthy participants and extends that research by making the novel finding that monetary losses associated with intentions can also improve PM performance. The generality of the finding suggests that value-added intentions have a general processing advantage. As mentioned earlier, Brandimonte et al. (2010) investigated the value of intentions in healthy adults and found that internal social rewards but not external rewards (in terms of additional partial course credit) improved PM. This discrepancy might be due to the different type of value-manipulation used in our study. That is, participants might value our monetary losses and gains higher than course-credit rewards. Furthermore, in our studies we clarified via instructions that *every* (in-)correct PM response would proportionally affect the final outcome. Brandimonte et al. however, were less specific in this regard by just telling participants that they would receive extra credit when they remembered to perform the PM task. Thus, their participants may have assumed that they would not receive the extra credit after having missed one cue and this could have reduced their motivation to respond to cues from then on. After all, rewards can influence goal-setting differently when allocated according to a piece-rate system with an opportunity to earn partial rewards regardless of overall goal achieved compared with a bonus system that is contingent only upon meeting a specified benchmark (Mowen et al., 1981). They also found that the outcome also interacts with the difficulty of achieving the goal such that effort increases with task difficulty for piece-rate rewards, but decreases for bonus-based rewards. This finding may account for the differences between studies, especially if our task was more demanding than that used by Brandimonte et al. (2010). This same pattern of results, however, might not hold for intentions associated with avoiding losses or late fees.

The finding that value-added intentions have a higher likelihood of being fulfilled than regular intentions is of high practical relevance. First, it is empirical support that late fees for PM failures (e.g., additional charges for late payments) or incentives for on-time PM fulfilment (e.g., health-insurance refunds for having a dentist check-up once a year) can indeed improve PM performance and apparently even without causing greater costs to one’s ongoing tasks. A practical implication thus might be that a usage of PM-associated punishments and rewards is warranted especially in domains where PM failures are crucial or even fatal (Grundgeiger et al., 2010; Dismukes, 2012). Furthermore, investigating whether certain populations such as children (Zimmermann and Meier, 2006; Maylor and Logie, 2010; Kliegel et al., 2013) or older adults (Zimmermann and Meier, 2006; Kliegel et al., 2008) who tend to have difficulties remembering intentions also benefit from value-added intentions.

The present findings of value-induced PM improvements in a fairly demanding PM task (syllable intention) that were accompanied by a rather small numerical increase in costs that was not statistically reliable, and is thus unlikely to fully explain the observed PM improvements, is challenging for current theorizing of PM. According to the proposed pathways of the motivational-cognitive model (Penningroth and Scott, 2007), goal relevance can facilitate effortless PM retrieval and/or change the engagement in effortful PM processing. Based on our manipulations, the model would predict that both the gain and loss frames could lead to *early* processing changes in the intention encoding that could result in changes to strategy use or to the accessibility of intentions, both of which would foster effortless intention retrieval. Alternatively, their model predicts the frames could lead to *later* processing changes in the allocation of attention to the PM task, which would always lead to higher costs to the ongoing task. When placed in the context of this model, improved PM performance for our frame conditions that were accompanied by neither substantially higher costs to the ongoing task (Experiments 1 and 2) nor by better recognition memory for neutral ongoing-task items (Experiment 2) appears to be partially consistent with the predictions of an effortless retrieval route. Based on our data from the Go/No-Go task of Experiment 2, we do not believe that the PM benefit was due to increased intention accessibility because we did not find any evidence that the presentation of PM critical items (i.e., items that served as PM cues in the preceding lexical-decision task) elicited faster go-responses or more commission errors in this test than neutral items in this test. Within the framework of the motivational-cognitive model (Penningroth and Scott, 2007) this leaves us with the final option that the value manipulation changed encoding-strategy use. There is some difficulty in assessing this explanation fully because Penningroth and Scott’s model does not specify which kind of strategy increased PM motivation should elicit. Finally, the fact that the perceived relative and absolute PM task importance was comparable between the loss-frame and the no-frame conditions further challenges the model, because a basic assumption of the model is that value-induced processing changes should be due to changes in perceived importance of intention fulfillment. The present results suggest, however, that intention-importance changes cannot fully explain the present results.

Nevertheless, we find the goal-based motivational-cognitive model to be a fruitful perspective on studying the dynamics of PM. Importantly, this model is the only motivational model proposed to explain motivational influences in PM. The model makes some clear predictions, which are also supported by data. Other aspects of the model are not as clear. Especially, the model does not consider that intention value could affect global attention allocation policies that are assumed to be established early during intention encoding based on metacognitive beliefs about task demands (Meeks et al., 2007; Rummel and Meiser, 2013; Rummel et al., 2013). Further, the dual-task nature of intentions suggests that attention-allocation policies may cause global changes in attention allocated to the entire task set or specific changes to the components of the task set. Marsh et al. (2005) have already made this argument for how the task demands might influence how participants allocate attention to the entire PM-task/ongoing-task set. The goal-based motivational-cognitive model only indicates attention allocated to the PM task. We understand that Penningroth and Scott (2007) believe that attention is already allocated toward some ongoing task and that their view of attentional allocation relates specifically to how attention is re-allocated toward the PM task and away from the ongoing task. Arguments for these attentional tradeoffs are rooted in theories of effortful processing and limited available resources (Navon and Gopher, 1979; Cowan et al., 2005) with which we generally agree. If our framing manipulations increased attention toward the PM task only, and that attention was allocated away from the ongoing task in favor of the PM task, we would expect to find attentional tradeoffs in terms of increased PM-induced costs. We did not, however, find such tradeoffs in either experiment. What is missing from the model is specification about how attention is allocated toward the overall task set (cf. Marsh et al., 2005).

Even though there might have been some changes in the recruitment of attentional resources for words processed in the framing conditions compared with the control condition, these changes are unlikely to completely explain the increased PM performance in the framing conditions because they were small. Further, they were not statistically reliable despite a good statistical power, which was higher than the power for the (significant) PM-performance comparisons. Thus, an alternative interpretation of our data is somewhat predicated on the possibility that attention allocated by participants in the no-frame control condition undermined their full potential, whereas the framing manipulations facilitated the recruitment of additional attentional resources that were otherwise not recruited by control participants.

The foregoing analysis highlights the possibility that certain value-added intentions may motivate participants to allocate more attention to the overall task set. Greater allocation of attention to the entire task set rather than to one component task could explain the increase in event-based cue detection in the framing conditions paired with the absence of a compensational increase in task interference. Perhaps clarifying the monetary promise as we did in Experiment 2 caused improvements for all conditions in that experiment. Although we do not have data to suggest that participants in our value-frame conditions allocated more attention toward the entire task set than those in the no-frame condition, the benefit for value-added intentions in the absence of attentional tradeoffs is very intriguing.

A limited understanding of motivational influences on PM warrants connecting our results with other research areas that have studied motivational mechanisms related to attention. A distinction between actual motivation and potential motivation is not new (Brehm and Self, 1989). According to the theory of motivation intensity, various determinants can affect the effort, or* potential motivation*, one is willing to allocate in order to satisfy a motive and that motivation “is created by needs and/or potential outcomes and the expectation that performance of a behavior will affect those needs and outcomes” (Brehm and Self, 1989, p. 111). In our experiments, the value frames represent the determinants that can influence motivation. Similar findings have been shown in reward domains (Mowen et al., 1981; Marien et al., 2014). In a recent study, Marien et al. (2014) used small monetary rewards for trials in a modality-shift paradigm and found evidence for changes in potential motivation when the difficulty of the task called for changes in adaptive control. Their effect was larger on crossmodal trials (e.g., visual-auditory) for which participants directed attention to a different modality than on ipsimodal trials (e.g., visual-visual). Others have also shown that motivational incentives and adaptive control can engage proactive control and brain activity in the prefrontal cortex and reward-related regions (Locke and Braver, 2008; Chiew and Braver, 2011; Marien et al., 2013) and have linked adaptive control to PM (cf. Braver, 2012), suggesting its importance in executing intentions.

An attentional shift and activation of the components of an intention may be vital for responding to PM cues (West and Craik, 1999; Marsh et al., 2002) which may be moderated by some form of adaptive control. In the absence of unambiguous experimental data, we are careful not to claim that a change in potential motivation accounts for our findings, but because of the similarities between attentional shifting in the modality-shift paradigm and that of detecting cues embedded in a PM task, we suggest this form of adaptive control is a testable explanation for future research.

Motivation appears to influence the completion of intentions in various ways. We used the goal-based motivational-cognitive model to help understand the role of motivation in the context of value-added intentions. Our data are both consistent and inconsistent with this model’s predictions. Unlike other models of PM that have been scrutinized and modified after ensuing years of scientific rigor, this model has not been tested to the same degree. In light of our findings, there may be some modifications of the model that might allow for it to better account for various types of data and intentions. We do, however, realize that our considerations to the model have not been scrutinized and are tentative. After all, our interpretations are limited to a subset of experiments designed to examine the influence of a 4€ gain or loss frame associated with detecting PM cues that we embedded in the context of word-nonword ongoing activities for an event-based intention. The literature on PM is vast and contains many different forms of intentions (e.g., activity-based, event-based, time-based), which may differ in processing demands and may be influenced differently by monetary gains or losses. Nevertheless, we have replicated and extended the research on incentives in the domain of PM and believe that fruitful avenues for investigation exist for both basic and applied domains of inquiry on these *value-added intentions*, which undoubtedly affect each and everyone one of us.

## Author and Contributors

The first two authors contributed equally to the present research. All three authors contributed substantially to the conception of the study, the data analysis, and interpretation of the data. SD supervised data collection. JR and GC drafted the manuscript and SD revised it. The final version of this article was approved by all authors.

## Conflict of Interest Statement

The authors declare that the research was conducted in the absence of any commercial or financial relationships that could be construed as a potential conflict of interest.

## Footnotes

^1^Although interesting questions could be asked about receiving payment vs. not receiving payment, we decided to pay all participants in the experiments in order to control for any effects of how payment itself changes the approach to the task(s) (Heyman and Ariely, 2004). Thus, any effects of our manipulation cannot be caused by the payment itself but by the increase in the value of the intention.

^2^In line with Penningroth and Scott (2007) motivational-cognitive model, we assumed that our loss manipulation may increase the perceived importance of the PM task, which, in turn, may result in an increase in cue monitoring at a cost to the ongoing task. In previous studies, instructions stressing the importance of the PM task yielded medium-sizes cost effects (e.g., Smith and Bayen, 2004; Loft et al., 2008; Loft and Humphreys, 2012). Therefore, we assumed an effect of medium-size in the population (i.e., *f* = 0.25; Cohen, 1968) for the present calculation of statistical power. Furthermore, because the correlations between baseline RTs and PM-block RTs were *r* > 0.70 in the two experiments reported here, we set the correlation among repeated measures to 0.70 for the power analyses for costs in both experiments.

^3^Although conventional significance testing suggested a reliable difference between conditions, the Bayesian analysis suggests that this difference was rather anecdotal (i.e., BF was smaller than 3; Jeffreys, 1961). However, we found reliable support for our hypothesis that a loss frame improves PM from both conventional and Bayesian analyses in Experiment 2 with an improved method (see below). Therefore, we believe that this effect is trustworthy.

^4^For within-subjects effects and between/within-subjects interactions, calculations of Cohen’s *f*, which was originally defined for between-subjects designs, are inflated estimates of the true effect size in the population when applying the usual conventions suggested by Cohen (1968). This is due to the fact that inter-individual variance is controlled for in within-subjects error terms (Erdfelder, personal communication). We therefore report $f_{z}=\sqrt{\left(f^{2}\times \frac{N-k}{N}\times \frac{m-1}{m}\times \left(1-r\right)\right)}$ as effect-size estimates for all within-effects and between/within-subjects interactions. Note that *k* is the number of levels of the between-subjects factor, *m* is the number of levels of the within-subjects factor, and *r* is the correlation between repeated measures. Effect-sizes *f*_z_ can be interpreted according to the usual conventions that refer to the between-subjects error term (i.e., *f*_z_ = 0.25 would indicate a medium-sized effect; Cohen, 1968).
